# Aurora-Trinity: A Super-Light Client for Distributed Ledger Networks Extending the Ethereum Trinity Client

**DOI:** 10.3390/s22051835

**Published:** 2022-02-25

**Authors:** Federico Matteo Benčić, Ivana Podnar Žarko

**Affiliations:** Faculty of Electrical Engineering and Computing, University of Zagreb, 10000 Zagreb, Croatia; ivana.podnar@fer.hr

**Keywords:** scalability, decentralization, light client, blockchain, trustless, Internet of Things

## Abstract

Light clients for distributed ledger networks can verify blockchain integrity by downloading and analyzing blockchain headers. They are designed to circumvent the high resource requirements, i.e., the large bandwidth and memory requirements that full nodes must meet, which are unsuitable for consumer-grade hardware and resource-constrained devices. Light clients rely on full nodes and trust them implicitly. This leaves them vulnerable to various types of attacks, ranging from accepting maliciously forged data to Eclipse attacks. We introduce Aurora-Trinity, a novel version of light clients that addresses the above-mentioned vulnerability by relying on our original Aurora module, which extends the Ethereum Trinity client. The Aurora module efficiently discovers the presence of malicious or Byzantine nodes in distributed ledger networks with a predefined and acceptable error rate and identifies at least one honest node for persistent or ephemeral communication. The identified honest node is used to detect the latest canonical chain head or to infer the state of an entry in the ledger without downloading the header chain, making the Aurora-Trinity client extremely efficient. It can run on consumer-grade hardware and resource-constrained devices, as the Aurora module consumes about 0.31 MB of RAM and 1 MB of storage at runtime.

## 1. Introduction

Distributed Ledger Technology (DLT) is one of the most striking discoveries in the field of distributed systems and digital asset management in recent years. DLT enables the management of a Digital Ledger (DL) in a distributed environment where participating peers do not trust each other. Such a DL is immutable, fault tolerant and replication transparent. The most popular specialization of the technology is the Blockchain with its pioneer Bitcoin, where data are stored in blocks in the form of transactions. The subsequent major breakthrough offered by public and permissionless solutions, such as Ethereum, is the ability to execute arbitrary code within smart contracts [1]. This allows the technology to be used in a variety of areas, including healthcare, communication systems, decentralized finance, electronic voting, and the Internet of Things (IoT) [2].

The peers in ledger networks can generally be classified as full nodes or light clients. Full nodes synchronize with the network by downloading the entire ledger to check the transaction history since the ledger genesis, while light clients store only a small portion of the ledger and are able to validate the ledger state and inquire about executed transactions. They do not interact directly with the ledger because they use full nodes as intermediaries, which sacrifices security for scalability. The features of light clients are attractive to end users, as consumer-grade hardware generally cannot meet the requirements for running a full node. In this paper, we focus on light clients, although the solution is generic and can be applied to full nodes as well.

Initial synchronization with the network can take a long time due to ledger size and its continuous growth [3]. In January 2020, the Ethereum ledger size in non-archived mode was about 250 GB [4], and its header chain size was 5 GB [5], with the header chain growing at the rate of 1 GB per year [6]. This compels users to use centralized block explorers and online wallets instead of running their own clients, sacrificing the decentralization property inherent to public and permissionless distributed ledger solutions, as users consequently have to rely on and trust third-party services.

The synchronization problem is exacerbated when considering the possible presence of malicious or Byzantine nodes in DLT networks, whose attacks range from denial-of-service to Eclipse attacks [7]. For example, a light client may face denial of service from a full DLT node, or from many full nodes forming a malicious clique, if a full node simply ignores its transaction inclusion or transaction submission request. Moreover, the process of initial header chain download can be regarded as a point of centralization, since it depends on a set of well-known nodes (i.e., addresses) being both available and not exhibiting malicious behavior. If these nodes exhibit malicious or Byzantine behavior, a new node entering the network may find itself completely surrounded by malicious nodes and remain isolated from the rest of the network, falling victim to an Eclipse attack. In the best case, after examining the downloaded header chain, the client will discover that part of the chain has been tampered with. Thus, it will waste its network bandwidth, time, and processing power before discovering an honest node, if there is one that can be reached by the client. In the worst case, there may be no honest nodes reachable by the client, and the client will fail to discover the canonical chain (the main chain recognized by the network as the source of truth) and may even accept a shorter chain as truthful.

One strategy that can protect a client from the aforementioned synchronization problems is to use Aurora, an algorithm designed to offer trustless verification and synchronization for distributed ledger networks [8]. The algorithm is stochastic in nature and enables a client to detect if it is being held up in a malicious clique with a low likelihood to make an erroneous decision. Thus, the client can make an informed decision whether to halt or continue its interaction with the ledger nodes. In other words, the client identifies at least one honest node or the presence of an Eclipse attack or network partition. The honest node(s) is (are) subsequently queried either to check transaction inclusion within the ledger or for header chain synchronization. The key difference between our algorithm and other state-of-the-art solutions is that the Aurora algorithm discovers remote nodes in a DLT network and asks nodes to reveal a list of their neighbor nodes to analyze the collected neighbor lists, unlike existing approaches that analyze chain integrity. Note that an overview of the algorithm is given for completeness of this paper in Section 3. Further details and a theoretical analysis of the algorithm can be found in [8,9].

In this paper, we introduce Aurora-Trinity, our extension of the Ethereum Trinity client (all statements regarding the client refer to a fork from Trinity v0.1.0-alpha.34 ‘Caroline Herschel’, commit SHA 7ebac95d) that integrates a module implementing the Aurora algorithm (source code is available at https://github.com/boorac/Trinity-Aurora-Client, accessed on 11 February 2022). The Aurora-Trinity client is used to investigate two important features of the Aurora algorithm in a real-world environment. First, the client should halt its operation and exit the network during an initial synchronization process if the existence of a malicious clique is detected. Second, the Aurora-Trinity client is expected to use fewer resources during transaction verification compared to state-of-the-art light clients because it does not need to download the header chain. A major advantage of Aurora-Trinity compared to other light client implementations is that it does not require changes of the consensus protocol or the introduction of additional data structures within ledger nodes.

This work builds on our previous work which introduced the Aurora algorithm [8] and now focuses on an actual client implementation and testing of the Aurora algorithm in a real-world environment, i.e., the client interacts with full nodes of the Ethereum mainnet. The main contributions of the paper are twofold:

- We implement the Aurora algorithm by extending the open-source Ethereum client Trinity. The Aurora-Trinity client provides the following new features:
  − It downloads a canonical chain head from an honest node, if Aurora can identify at least one honest node with high likelihood. This simplifies header chain synchronization in the presence of malicious network nodes and consequently reduces the resource consumption of a light client.
  − The client uses an efficient method to verify the presence of a transaction in a ledger by querying honest nodes. The method does not require the download of header chain or even its part from full nodes.
- We evaluate our implementation in the Ethereum mainnet and propose further improvements for the mainnet protocols.

The remainder of this paper is organized as follows: Section 2 discusses related work. In Section 3, we provide an overview of our model, algorithm and highlight its added value in the presence of malicious or Byzantine actors in ledger networks. Section 4 describes Trinity, an open-source client for Ethereum, enumerates Ethereum network properties relevant to the implementation of our algorithm and presents the architecture of the Trinity client after it was modified to include the Aurora module. Section 5 presents the experimental evaluation setup and the results of the execution on the Ethereum mainnet, followed by a comparison with other Ethereum clients. Section 6 reflects on the collected results and opens a discussion for future work, while Section 7 concludes the paper.

## 2. Related Work

We begin with a brief survey of the work dealing with adversarial influence and countermeasures in P2P networks, with focus on DLT networks. Admittedly, our work is not directly comparable to this category for three main reasons. First, rather than attempting to detect a specific malicious node and to mitigate the direct impact of malicious nodes, we circumvent the problem by detecting an honest node for ephemeral or persistent communication. Second, our work is domain-specific, i.e., it relates to bootstrapping and transaction inclusion verification in DLT networks, and does not focus on a general problem of identifying malicious nodes in P2P networks. Third, our algorithm is not necessarily useful only in the presence of an Eclipse attack, but also in the absence of an Eclipse attack when malicious nodes expose malicious data without mutual coordination. Nonetheless, the presentation of this problem strengthens our claim that DLT clients are vulnerable to malicious nodes, which reinforces the need for Aurora-enabled DLT clients.

The influence of adversarial nodes in P2P networks has been thoroughly researched in [10,11,12], with recent work focusing on DLT networks such as Ethereum [13,14,15,16,17] and Bitcoin [7,17,18]. The identified problems are diverse and include, but are not limited to the following: Eclipse Attacks, DoS and DDoS attacks, manipulation of routing advertisements (i.e., BGP hijacks), man-in-the-middle attacks and Sybil attacks (a recent study reported a Sybil attack that was executed on the Bitcoin Cash network with up to 5000 Sybil nodes [19]). They may lead to serious consequences, e.g., synchronizing with a longer chain with lower total difficulty in Proof of Work (PoW) networks, double spending, filtering the victims view of the ledger, co-opting the victim’s computing power, etc.

We continue by comparing the Aurora-Trinity client to other existing DLT clients. Hereinafter, we classify Light DLT clients into three categories, based on their trust model and the amount of storage they require: remote clients, Simplified Payment Verification clients, and super light clients.

Remote clients rely almost entirely on the honesty and availability of their gateways, unlike the Aurora-Trinity client that does not depend on any single entity. As such, these clients implicitly trust their gateways, which makes them trusted (the opposite of trustless), have very low hardware requirements in terms of storage and, for this reason, are convenient for an end user, but at the cost of DLT’s security and decentralization properties. An example of such client is MetaMask, a wallet for Ethereum that communicates with the ledger via the Infura Gateway [20]. A variation of this approach is an architecture where multiple gateways are used instead of a single gateway. An example is SlockIt INCUBED Client (Slock.it Incubed3|ConsenSys Diligence—https://consensys.net/diligence/audits/2019/09/slock.it-incubed3/, accessed on 11 February 2022). The approach uses an incentive scheme where multiple gateways connect to the ledger and offer a stake as a guarantee for honest behavior.

Simplified Payment Verification clients (SPVs) [21] download a portion of the ledger to verify the state of the ledger, e.g., to check whether funds are held by an address, or to verify the inclusion of a transaction within a ledger. As such, they can be regarded as trustless. SPVs download the header chain instead of the entire chain, and are able to request metadata from a full node on which they depend to verify the content of the ledger. Compared to remote clients, they are less dependent on their respective gateways, but they still rely on full nodes to function. Examples of such clients are Geth in light mode and Electrum (FAQ—Electrum 3.3 documentation—https://electrum.readthedocs.io/en/latest/faq.html#how-does-electrum-work, accessed on 11 February 2022), a solution that connects to multiple gateways when subscribing to block header updates, which supports our premise that multiple sources of truth increase security.

Finally, super light clients also download a portion of the ledger, but usually a smaller portion compared to SPVs, while trying to maintain the trustless property of DLT. Our client fits into this category, as it is trustless and requires less storage compared to SPVs. A solution proposed in [22] uses a cryptographic accumulator to generate a new block attribute, a chain summary, which is maintained by all peers and requires changes of DLT software and protocols. A client randomly selects a subset of DLT nodes to verify the chain state using the chain summary. If the majority of the selected nodes are compromised, the protocol is not secure, which means that this solution could be used in conjunction with the Aurora algorithm to identify a required number of honest nodes for future interactions. Another super light client is proposed by Vault [23] that builds on the Algorand consensus protocol and introduces a fast bootstrapping method relying on the existence of additional data structures (namely, stamping certificates). It contrasts with our solution which does not require the introduction of additional data structures. The following two solutions also require the maintenance of additional data structures: NIPoPoWs [24] and FlyClient [25] are Proof of Work (PoW) specific solutions that require the client to store only a logarithmic number of block headers instead of the entire header chain. FlyClient uses probabilistic sampling, the Fiat–Shamir heuristic, and introduces a new data structure, Merkle Mountain Ranges (MMRs). Most importantly, the client must be connected to at least one honest node—a node that can be provided by our algorithm. One solution that is resilient to Eclipse attacks is BlockQuick [26]. It achieves resilience by using a consensus-based reputation scheme, but again at the cost of introducing additional data structures. In summary, all of the above solutions primarily use information from the data stored in the ledger itself to operate in a trustless environment, while the Aurora algorithm adds another layer of trust into DLT solutions by relying on the analysis of neighbor lists that other remote nodes reveal.

## 3. Network Model and Aurora Algorithm

In this section, for completeness, we introduce and justify the assumptions, introduce the network model, and then provide an overview of how the Aurora algorithm works and include the associated pseudocode. The model and algorithm are fully defined in [8].

### 3.1. Assumptions and Network Model

Let us assume that an honest node *a* new-node-identifier enters a DLT network. The goal of node *a* is to discover an honest ledger node for ephemeral or persistent communication. The DLT network is modeled under the following assumptions:

**Assumption** **1.** *The majority of network nodes are honest. This is a standard assumption in DLT networks [21,27,28]. Bitcoin, for example, assumes that the honest majority constructs the strongest chain, a rule that accounts for the cumulative difficulty of the Proof of Work puzzle [29]. Proof of Stake protocols assume that the honest majority will stake the majority of network tokens as a guarantee of honest behavior [28].*

**Assumption** **2.** *Peer information and ledger metadata are exchanged between network nodes. This is a standard assumption in DLT networks [8,27,28]. The new node *a* can query other remote nodes for their neighboring peers using an active-peers request message (apreq). The queried nodes respond with an active-peers response messages (apres). Furthermore, node *a* can ask for the canonical chain head by sending a head message to a full node. Finally, node *a* can request a proof that a transaction is included in a ledger block by sending a proof message. We map our abstract messages to the actual messages in protocols supported by Ethereum in Section 5.*

**Assumption** **3.** *Bootnodes may be unavailable or malicious. As a result of adversarial influence or failures explained in Section 2, the bootnodes needed by the new node to discover the network may be unavailable or malicious. This problem, called fake bootstrapping, has been explored in the literature [30]. Bitcoin, for example, recognizes and addresses this problem by using cached peers for subsequent connections, employing a pseudo-random protocol to obtain a subset of potential bootstrapping peers, using DNS to query remote nodes for other peers, and using a list of hardcoded nodes when the nodes behind DNS are unavailable. Nevertheless, malicious influence is still possible with all of the above mechanisms [31].*

**Assumption** **4.** *Bootstrap candidates can eventually be discovered. This is a standard assumption in DLT and P2P networks. A bootstrap candidate can eventually be found, e.g., using the Random Address Probing method [30], or through social contacts, news channels, or the like. Such bootstrap candidates can be contacted manually by node *a*. Bitcoin clients, for example, provide a special remote procedure called addNode to connect to other clients (https://developer.bitcoin.org/reference/rpc/addnode.html, accessed on 11 February 2022).*

Network Model: Under the stated assumptions, a DLT network is composed of a set of network nodes *S* that has a size of |*S*|, where the network may contain a set of malicious nodes *L*, which has the size of *l*. Node *a* is aware of at least one node *s*_0_ that can be used for bootstrapping (the solution is easily extendable to multiple nodes) that may be malicious and attempt to subvert node *a*. Information about remote peers and ledger information is shared between the nodes using the following messages: apreq, apres, head, and proof.

The Adversary: An adversary is a member of the set of malicious nodes *L* that exhibits Byzantine or malicious behavior to subvert the node *a*. We assume that malicious nodes tend to form a clique, as observed in [32], and that malicious nodes choose to expose other malicious nodes as their neighbors to prevent the node *a* from contacting honest nodes. Honest nodes, on the contrary, disclose their neighbors randomly (i.e., according to the protocol). When a malicious node receives a head or a proof message, the malicious node can choose to spoof a response or not respond at all, unlike honest nodes that respond to both messages honestly and in accordance with the protocol.

### 3.2. Aurora Algorithm

Under the above assumptions, a light client executing the Aurora algorithm exchanges a series of peer discovery messages with nodes on a DLT network to determine with a quantified likelihood that the client, i.e., a new node joining the network, is exposed to a malicious clique. When the presence of such a clique is detected, the algorithm either provides an honest node for ephemeral or persistent communication [8] or halts further operation of the client. By using the Aurora algorithm, the client is able to protect itself from synchronizing with nodes that expose a non-canonical or forged chain (when the client acts as an SPV). In addition, the algorithm can be used to verify the inclusion of transactions in a given block with high likelihood without downloading the entire chain or header chain (when the client acts as a super-light client). Since this paper focuses on the implementation and integration of our solution into an existing DLT client, we refer the reader to [8] for a more detailed and formal description of the algorithm, and provide an extended summary of the algorithm herein.

A light client recursively discovers nodes on a DLT network via the Aurora algorithm and in a manner that can be depicted as the generation of a Directed Acyclic Graph (DAG) on the network topology as part of a process called a walk. Similar to Ethereum’s boot process, each step of the walk randomly chooses an unvisited node from the peer pool (a set of known nodes) and sends an apreq message. The nodes queried about their neighboring peers respond with an apres message, whereupon the peers contained in the apres messages are added to the client’s pool of known nodes. A walk is executed in a series of discrete steps, each step being referred to as a hop. A hop includes sending an apreq message to a full node and receiving an apres message from the full node.

**Figure 1:**
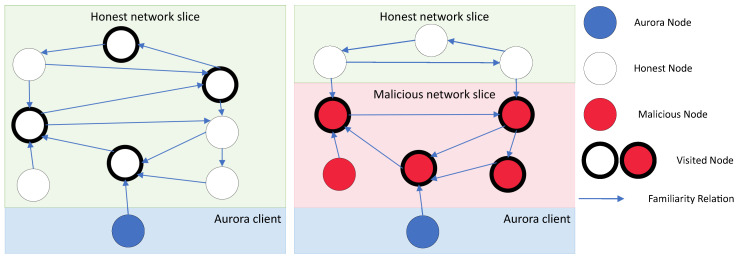
The figure on the left shows a client running the Aurora algorithm as it traverses the honest part of the network. The right figure shows the client traversing a malicious clique.

The client continues with a walk until one of two mutually exclusive events happen. First, the maximum number of hops which is defined by a specific parameter *d* has been reached within the walk. After *d* hops, the walk ensures to end at an honest node that will be used for future communication (see Figure 1). Second, the client identifies that nodes omit to report new nodes in their apres messages and terminates the bootstrap process since such behavior signals the presence of malicious nodes.

As a result of the adversary’s behavior, a new node entering the network would not discover an honest network node, and thus would not be able to discover a canonical chain maintained by the honest majority of nodes.

The distance parameter is calculated to execute enough hops such that a client’s pool of known nodes contains more nodes than the assumed number of malicious network nodes. In other words, at least one honest node is included in the pool of known nodes if the pool includes more nodes than the assumed number of malicious nodes which is ⌈|*S*|/2⌉ − 1 (this is a conservative default number of malicious nodes).

**Figure 2:**
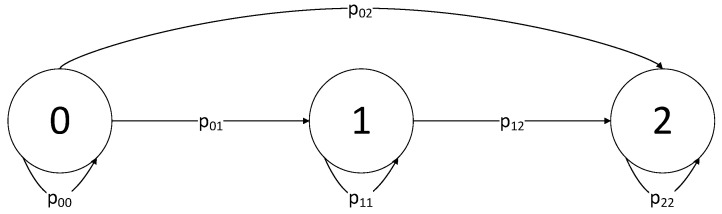
Markov chain with state space E={0,1,…l = 2}. Each state corresponds to the number of distinct nodes that exist in the pool of known nodes of the client, e.g., state 1 means we found 1 out of 2 nodes.

The parameter *d* is computed using Markov chains [33]. We construct a Markov chain with a state space of size equal to the assumed number of malicious nodes E={0,1…l=⌈|S|/2⌉ − 1} (an example is given in Figure 2), where each state corresponds to the number of unique nodes that exist in the pool of known nodes of the client. The transition probabilities of such a chain are derived from the hypergeometric distribution probability mass function:

(1) P(K=k)=mkN−mn−kNn=f(N,m,n,k)

We then construct a transition matrix *PM*, where state *l* is absorbing, meaning $PM_{ll}=1$:

(2) $$PM=\left[\begin{array}{ccc} p_{00} & \cdots & p_{0l}\\ \vdots & \ddots & \\ \; & \; & p_{(l-1)l}\\ 0 & 0 & 1 \end{array}\right]$$

where the members of the matrix $p_{ij}$ are calculated using Equation (1):

(3) $$p_{ij}=\left\{\begin{array}{cc} 0, & \mathrm{if}\;i>j\\ f(\left|S\right|,l-i,\bar{\left|\mathrm{APRES}\right|},i-j), & \mathrm{otherwise} \end{array}\right..$$

We now define the matrix Y as a l∗l sub matrix of *PM* without the rows and columns of absorbing states, so that the matrix contains only transient states. Then, we calculate the matrix $\Pi ={\left(I-Y\right)}^{-1}$. If we denote $E[\Pi_{l}]$ as the expected number of hops before we discover *l* malicious nodes, it can be computed by Equation (4).

(4) $$d=E\left[\Pi_{l}\right]=\sum_{j=0}^{l-1}\pi_{0j}$$

Simply put, the distance parameter *d* represents the expected number of hops that the client must execute in order for the pool of known nodes to contain *l* malicious nodes. If no suspicious behavior was detected during the walk, the next newly discovered node is considered honest.

Suspicious behavior of nodes encountered during a single walk is measured relative to a threshold value *λ*, which is an input parameter of the algorithm expressing the user’s tolerance to suspicious behavior, where a lower *λ* represents lower tolerance and vice versa. The value *λ* is a real number 0≤λ≤∞ and can be arbitrarily set by a user.

After the end of each walk, the node executing a walk queries the detected honest peer for the current chain head with a head request. The chain head received in response to a head query is associated with an indicator which signals that a walk ended at an honest node, called a correctness indicator *z*, 0≤z≤1.

(5) $$z=1-\frac{\alpha }{\lambda };\;\;0\leq \alpha \leq \lambda$$

where *α* quantifies suspicious behavior relative to *λ*. If the correctness indicator *z* ever reaches 0, the client decides that remote nodes are intentionally omitting to report new nodes in their apres messages, which is considered suspicious behavior, and as a result, the client terminates. If *d* hops were made before *z* reaches 0, the algorithm outputs the identifier of an honest node that can be queried to verify whether or not a particular transaction was included in the chain, by not only querying the peers with a head request, but also simultaneously requesting the proof (Merkle proof for a transaction claimed to be included in a block), thus eliminating the need to download a header chain if the responding peer is honest.

In terms of complexity, our client performs two different operations: it computes the distance and performs a *walk*. First, to compute *d*, the client must construct *PM* defined in Equation (2) containing ${\left(l+1\right)}^{2}$ members, then to extract Y containing $l^{2}$ members, then the matrix Π containing $l^{2}$ members is calculated to derive *d*, as defined in Equation (4), which requires the sum of *l* members. Therefore, the total number of operations performed to compute *d* depends on the assumed number of malicious nodes and can be expressed as follows:

(6) $${\left(l+1\right)}^{2}+2*l^{2}+l=3l^{2}+3l+1$$

Second, a walk is performed. It stops when one of two mutually exclusive events happens: either at most *d* hops were made or *z* has reached 0. Consequently, the longest walk happens when *z* does not reach 0 (which happens when λ approaches *∞*) and a maximum of *d* hops are made. Using Equation (4), the total number of messages a client will exchange before first discovering an honest node and then querying the latest ledger state or the state of a transaction can be expressed as:

(7) $$2*d+2=2*\sum_{j=0}^{l-1}\pi_{0j}+2$$

More precisely, if $\sum_{j=0}^{l-1}\pi_{0j}$ hops are made during one walk, twice the amount of messages was exchanged, because a single *hop* consists of sending one apreq and receiving one apres message. After the end of a walk, two more messages are exchanged, either a *head* request and a response or a *proof* request and a response.

Based on the solution description given here, a simplified pseudocode of the algorithm is given in Algorithm 1, and is further elaborated in the context of Ethereum in Section 4.3. First, the variable *d* is calculated (line (1)). Second, the first contact node *s*_0_ is set as the target for the next hop, and the set of known nodes $S_{h}$ is initialized (lines (2–4)). In a loop, apreq and apres messages are exchanged and the contents of the apres messages are used to update α (lines (6–8)). A random, uncontacted node from $S_{h}$ is used for the next hop (lines (11–12)). If *d* hops were made (line (5)), or *z* reaches (0) (line (9)), the walk ends and the result is returned (line (14)).
**Algorithm 1** Aurora Algorithm Pseudocode.1:$d\leftarrow calculateDistance(\left|S\right|,\bar{\left|\mathrm{APRES}\right|})$2:$nxtHop\leftarrow s_{0}$3:i←04:$S_{h}\leftarrow \left\{s_{0}\right\}$5:**while** i<d **do**6:     Send APREQ to node nxtHop7:     P← APRES from nxtHop8:     Update α according to the amount of unique nodes in *P*9:    **if** α=λ **then**10:         **break**11:     $S_{h}\leftarrow S_{h}\cup P$12:     nxtHop← random node not already contacted from $S_{h}$13:z←1−α/λ14:**return** (z,nxtHop)

## 4. Trinity Client and the Aurora Module

Since we focus on light clients in this paper, we review distinguished DLT networks, such as Bitcoin and Ethereum, to identify the available light client implementations. On the one hand, MultiBit, Bitcoin Wallet, and Electrum are good examples of SPV clients for the Bitcoin network. On the other hand, for Ethereum, the official reference implementations are the following: Aleth (C++ ETH client: https://github.com/ethereum/aleth, accessed on 11 February 2022), Trinity (Python ETH client: https://github.com/ethereum/trinity/, accessed on 11 February 2022), and Geth (Go ETH client: https://github.com/ethereum/go-ethereum, accessed on 11 February 2022). Out of the listed three, Trinity and Geth provide the ability to run in light client mode. In the context of this work, Trinity was chosen as a suitable client for integrating the Aurora module due to its growing developer ecosystem, expressive code base, and popularity of the Python programming language.

Trinity is an open source Ethereum client implementation written in Python and built on top of a Python implementation of the Ethereum Virtual Machine (Py-EVM), with the goal of becoming a de facto standard for the Ethereum Python ecosystem. It is maintained by the Ethereum Foundation Python team and a broader Python Ethereum community. It has been in development since November 2016. Although the project is still in its relatively early stages, it includes all the features needed to integrate the Aurora algorithm, such as the ability to run clients in full and light mode, and has a Component API that allows developers to create components that easily extend the system functionality in a modular fashion.

The importance of Component API stems from the fact that it allows efficient and decoupled communication between different components of Trinity, which often run concurrently in different processes (Trinity Documentation—https://trinity-client.readthedocs.io, accessed on 11 February 2022). Concurrency is a necessity in the Trinity system since a client processes a continuous flow of events: It responds to peers, executes transactions, validates blocks, etc. The processes communicate by the use of a lightweight, multi-process event bus called Lahja. By using the Component API, we integrate our module into Trinity. The integration of the Aurora module in the Trinity client extends the client with the following features:

**Feature** **1.** *The client is able to detect malicious nodes with a certain likelihood, and, if a connection to an honest node can be established, synchronize with the network, or otherwise leave the network with a warning.*

**Feature** **2.** *The client is able to validate a transaction with a certain likelihood, in a network containing malicious nodes, without downloading the full ledger or header chain.*

Since the Aurora algorithm depends on both the peer discovery mechanisms and block header information, we provide an overview of the two main peer-to-peer network protocols of the Ethereum network, namely, the Node Discovery Protocol (NDP) (NDP: Node Discovery Protocol v4, with NDP v5.1 marked as work in progress at the time of writing) and RLPx Transport Protocol (RLPx) (RLPx: https://github.com/ethereum/devp2p/blob/master/rlpx.md, accessed on 11 February 2022), which facilitate peer discovery, the maintenance of routing tables and chain state.

### 4.1. Node Discovery Protocol

The Node Discovery Protocol (NDP) is used to discover nodes in the network via a Distributed Hash Table (DHT), and is inspired by and similar to the peer-to-peer DHT Kademlia [34]. NDP specifies messages needed to maintain a P2P network, such as ping-pong messages, messages including connectivity information, and node lookup requests. The Kademlia-like structure is used by Ethereum because it efficiently organizes a distributed index of nodes, and leads to a network topology characterized by a small diameter (devp2p—https://github.com/ethereum/devp2p, accessed on 11 February 2022).

Each Ethereum node stores a table of known active nodes. The table has at most (255) rows and contains *k* nodes in each row, where *k* is a network parameter which is not set globally, but usually k=16. A node adds a remote peer to the row in the table according to the distance of the remote peer to itself. The distance between peers in Ethereum is a bitwise XOR of the public key hashes, interpreted as a number. When a node is queried with a lookup request from a remote peer, it responds with the peer list made up of a row from its table corresponding to the remote peer (https://github.com/ethereum/devp2p/blob/master/discv4.md, accessed on 11 February 2022).

The recursive lookup is an iterative peer discovery process used by Ethereum nodes over the NDP protocol. First, a node initiating the recursive lookup selects *n* nodes closest to the referent node, sends a lookup request to them, and, after retrieving the responses, iteratively sends a lookup request to *n* nodes, but only to nodes that have not yet been queried. This continues until no more nodes can be found to populate the Kademlia-like table with newly discovered nodes. When a new peer joins the network, it runs a boot process. This is a recursive lookup process initiated with a set of default nodes called bootnodes to populate its Kademlia-like table with peers.

### 4.2. Rlpx Transport Protocol

RLPx is a TCP-based protocol used by Ethereum nodes to communicate blockchain-specific data with each other over the network. Blocks and block headers, Merkle tree roots, transactions, and transaction receipts are all examples of information that fall within the RLPx scope. RLPx defines two sub-protocols, the Ethereum Wire Protocol (ETH) (ETH—https://github.com/ethereum/devp2p/blob/master/caps/eth.md, accessed on 11 February 2022) and Light Ethereum Protocol (LES) (LES—https://eth.wiki/en/concepts/light-client-protocol, accessed on 11 February 2022). The LES protocol is used by Trinity when a client joins the network as a light client, and is therefore of great importance to the Aurora module, while the ETH protocol is used by full nodes. LES specifies requests relevant to Merkle proofs and header chain information, while ETH uses request relevant to block body and header chain information.

Since there are not enough incentives for a full Ethereum node to serve LES requests, only a small percentage of Ethereum mainnet nodes support LES. A study conducted between April and July 2018 reports that only 1.24% of Ethereum mainnet nodes supported LES [35]. This report correlates to our results which we elaborate in Section 5. Since the Aurora client depends on LES, this finding creates a significant problem, not only for the Aurora-Trinity client, but also for any other light client on the Ethereum network, which is out of the scope of this work.

Table 1 shows a mapping of abstract messages listed in Section 3 to the corresponding messages and protocol used by Ethereum clients, including Trinity. The NDP protocol message FindNode can be used to retrieve peer neighbors, the head hash information can be found in Status response messages (supported by both the ETH and LES protocols), while transaction proof can be fetched with GetProofsV2, which is used by the LES protocol. Since the transaction proof request can only be made using the LES protocol, Feature 2 can only be used when Trinity is running in a light client mode. Feature 1, however, can be used in both light and full modes.

### 4.3. Architecture of the Aurora-Trinity Client

**Figure 3:**
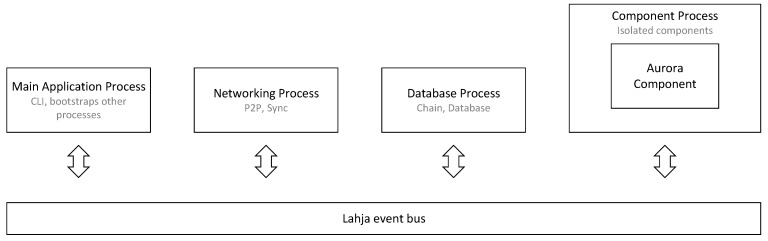
High-level representation of communication between Trinity components. The Main Application Process starts the Networking Process and the Database Process. The Networking Process handles peer-to-peer communication. The Database Process provides operations related to the chain and uses LevelDB by default. The Aurora Component enables the use of Feature 1 and Feature 2 on the client.

By using the Trinity Component API, the client is extended with a modular component implementing the Aurora algorithm to form a client with new capabilities. This component is hereafter referred to as the Aurora Component. The Aurora Component is spawned in its own isolated process and communicates with the rest of the Trinity system through the Lahja event bus. Data, such as peer neighbors, block header information, or transaction proofs from other peers are shared over the event bus. The Aurora Component requests this information by sending requests over the event bus, which are consumed by components assigned to provide answers to those specific requests. After delegated components broadcast a response, the Aurora Component can retrieve it from the event bus, as shown in Figure 3. During system initialization, the Aurora Component will replace the Trinity boot process, which is also capable of populating the peer pool with its discovery mechanism.

As a prerequisite for its execution, the Aurora algorithm needs a set arguments to be provided by an end user, namely:

- |*S*|: the assumed public and reachable network size;
- *s*_0_: identifier of the first peer from which a walk should start (in case the bootnodes used by default are not available);
- λ: a threshold estimating acceptable suspicious behavior;
- $\bar{\left|\mathrm{APRES}\right|}$: the average size of apres message containing peer identifiers. An identifier is made up of the secp256k1 public key, IP address and port of the remote peer. For Ethereum, this is set as a network-wide constant which usually equals 16 (https://eth.wiki/en/fundamentals/enode-url-format, accessed on 11 February 2022);
- *d*: the number of hops to be made before the algorithm should halt, derived transitively from the Markov chain analysis and |*S*|, and optimized at runtime, as defined in [8].

The listed arguments are sufficient to enable the execution of Feature 1. To enable Feature 2, in addition to the listed arguments, a user needs to supply a transaction identifier *tx_id_* to be verified and a block identifier *blk_id_* which includes transaction *tx_id_*. The second configuration runs the Trinity client without any synchronization mechanism in place. The outputs of such execution are a transaction inclusion proof and overall correctness indicator *z*.

Given that Trinity client was in the alpha version at the time of implementation of our client (at the moment of writing, the client remains in alpha—Trinity v0.1.0-alpha.36 ‘Lynn Margulis’), there were some setbacks. No API support for head and proof requests and responses over the event bus existed. The components handling the messages did exist, but the API for inter-process communication over the event bus did not, so the API had to be extended ad hoc for the Aurora-Trinity client implementation.

To extend the aforementioned API and integrate the required Aurora features into Trinity, we introduced changes to the proxy peer pool component, a component designed to be used from any process to interact with the peer pool data. At the time of the Aurora client implementation, parts of the Trinity client were refactored to use a different concurrency library (trio), that is incompatible with the one previously used (asyncio). As the proxy peer pool implementation was based on an asyncio service and the discovery component was based on a trio service management, we had to create a temporary workaround and implement the Aurora Component as part of the discovery component logic, rather than as an independent component. The main reason for this decision was the fact that the service exposing the API for peer discovery was merged with the discovery component, and it was not trivial to isolate them. Hence, the Aurora Component implementation is currently tightly coupled with the Trinity core code. However, when the Trinity API stabilizes, it will be possible to decouple the Aurora Component from other Trinity components so that anyone running a Trinity node can add Aurora features to the client by installing a Python package (https://trinity-client.readthedocs.io, accessed on 11 February 2022).

## 5. Methodology and Experiments

Methodology: In the scope of this section, we provide an answer to two specific research questions. First, can our solution operate in a production DLT network? Second, how much resources does our solution require while operating in a production DLT network? For this purpose, we introduced a full new node on the mainnet and executed walk until the correctness indicator *z* and the amount of RAM used by the client stabilized. Furthermore, a total of 4807 connections with other nodes were attempted and various algorithm outcomes were detected and classified. Note that both the initial ledger download as well transaction inclusion verification are read operations in the sense that they do not change the ledger state, i.e, none of these processes incur transaction fees because no transactions are issued. Moreover, our solution does not change the underlying consensus mechanism and does not affect the number of transactions per second that can be processed by the network. In the context of Ethereum, this means that the number of transactions per second the network can handle will remain roughly between 7 and 5 transactions per second regardless of the client in which our solution can be integrated (geth, Trinity, etc.) [28]. Furthermore, the integration of our solution will not influence transaction execution costs, i.e., the costs will still depend on the type of the transaction, state of the network and its congestion. For example, a simple transfer of Ethereum tokens will require a gas limit of 21,000 units of gas (https://ethereum.org/en/developers/docs/gas/#what-is-gas-limit, accessed on 11 February 2022, while the price of executing such a transaction in Ether fluctuates depending on network congestion, regardless of our solution).

**Figure 4:**
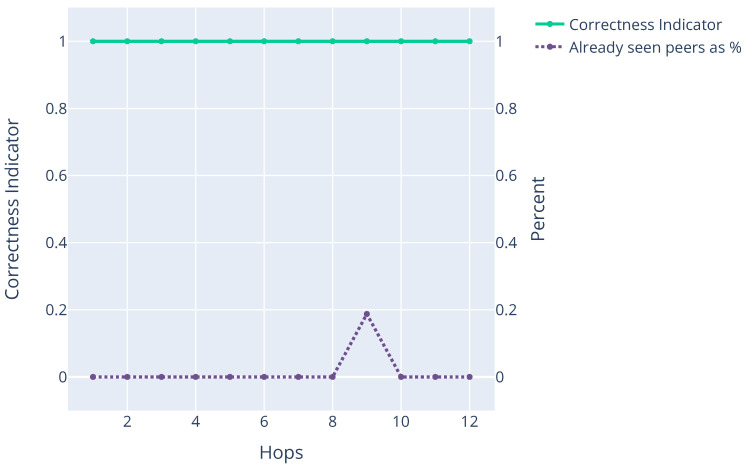
Example mainnet experiment when a small number of previously seen nodes were exchanged in apres messages as the number of hops increased.

The execution of walk on the mainnet provided a valuable insight into the issues that need to be addressed if the client is to be used in practical applications on the main Ethereum network. Figure 4 shows an example run on the Ethereum mainnet and consists of two traces. First, the trace “Already seen peers as (%)” shows the percentage of already seen peers in an apres message received per hop. Second, the Correctness Indicator trace shows the value of *z* as expressed by Equation (5). From these values, we can conclude that a small number of already seen nodes are present in apres messages, and the Aurora algorithm concludes that remote nodes do not intentionally omit new nodes from apres messages, which is normal in the absence of an Eclipse attack. We can thus conclude that our solution operates as expected in the absence of an Eclipse attack. However, we cannot conclude the opposite, as this would require introducing malicious nodes into the Ethereum mainnet (which is an attack on a production network) and knowledge of the network topology (Ethereum is an anonymous peer-to-peer system where peers can join and leave at their discretion, which we cannot influence). An alternative approach is proposed instead in Section 7.

Second, the Aurora paper [8] uses a simplified network model in which nodes are considered always available, meaning that a client can retrieve the head or proof from a selected node after network traversal is complete. However, in public networks, such as Ropsten or mainnet, this assumption is not always true. During the execution of multiple synchronizations with the mainnet network, various exceptions were thrown when the client attempted to connect to other nodes using the RLPx protocol. An overview of these exceptions is listed in Table 2. Since network clients typically only accept a finite number of connected peers at a time, executions on the Ethereum mainnet network often resulted in a failure to connect to a particular peer due to the maximum peer pool capacity.

The results also show that a large number of nodes does not support the LES protocol, as 64.24% of the connection requests failed because the protocol was not supported. This result correlates with the findings provided in [35]. Since this is more of a general network problem, there are not many client-side solutions to solve this problem. A partial solution would be to modify the algorithm to store the nodes discovered at each hop in a LIFO data structure that contains backup candidates in case of unsuccessful RLPx connection attempts. In the future, better support for the LES protocol is expected, as well as a smaller number of peers reaching the maximum peer pool capacity, which would lead to more successful connection attempts (see Discussion in Section 6).

**Figure 5:**
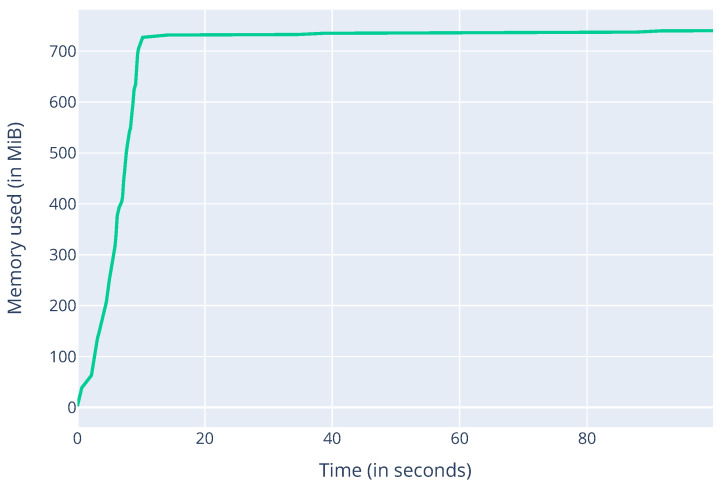
Memory consumption of the Aurora client in light mode measured with memory-profiler for Python.

**Figure 6:**
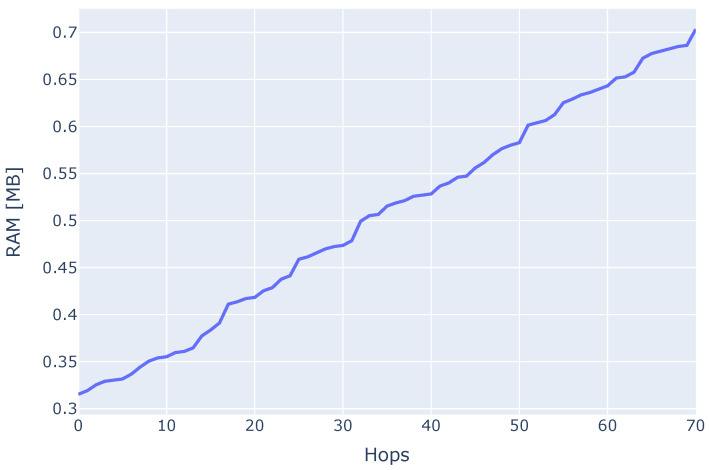
Memory consumption of the Aurora module measured with memory-profiler for Python.

Next, we measured the memory consumption of the Aurora-Trinity client in light mode with a memory-profiler for Python, as shown in Figure 5. The approximate (800) MB RAM requirement could be significantly decreased by removing all modules that are not necessary for running the Aurora algorithm and optimizing the client code overall. Note that this value is closely related to the implementation of the Trinity client. To better understand the resource consumption of the Aurora module, we performed a walk on the Ethereum mainnet so that the client had to collect 1000 nodes for its node-pool and measured the RAM consumption of the module within Trinity. The results are displayed in Figure 6 and show a steady increase in RAM consumption as the client discovers new nodes during a walk. Starting from about 0.31 MB when the execution starts and when no nodes are discovered, memory consumption increases to about 0.7 MB when 1000 nodes are discovered. Applying linear regression to the graph shown in Figure 6, we obtain the following expression: y(hops)=0.00565∗hops+0.307, which describes memory consumption in MB as a function of the number of hops. The number of Ethereum nodes discovered on 7 December 2021 (https://etherscan.io/nodetracker, accessed on 11 February 2022) was 2543. Assuming 16 peer identifiers are discovered per hop, 2543 nodes are collected in 159 hops, and using the above expression, our client would consume 1.2 MB.

A threat to using the Aurora algorithm in practice is the long walk execution time due to network latency. Given that the time required to execute each hop in a walk is on average 7 s for executions on the mainnet network (with 70 MB/s download and 15 MB/s upload network connection, the average time for each hop consisted mainly of network latency and processing requests from remote peers), the overall execution time of the Aurora algorithm on a larger network, such as the mainnet, is a point of concern. Assuming that the average time for each hop is 7 s, the $\bar{\left|\mathrm{APRES}\right|}$ is 48, and the assumed number of malicious nodes is 100, the execution time of the Aurora algorithm would be 44 min (see Table 3).

As a solution, execution time can be reduced by increasing the number of neighboring peers the client can discover in parallel. If *x* walks are made in parallel, then the time required to exchange the maximum number of messages before the algorithm terminates (examples are given in Table 3, column “walk duration”) is divided by *x*.

Note that the Bitcoin protocol supports apres message (called *ADDR* messages) with size up to 1000 (Bitcoin—Developer Reference—https://developer.bitcoin.org/reference/, accessed on 11 February 2022) node identifiers, which could drastically improve the execution time of Aurora in Bitcoin networks.

This occurs primarily because the increase in sample size *n* (which corresponds to the increase of $\bar{\left|\mathrm{APRES}\right|}$), as expressed in Equation (1), reduces the probability of sampling 0 new nodes, which would reach 0 if n=N. This, in turn, would reduce the sum represented by Equation (4) and as a consequence reduce *d*.

In addition to network latency, the computation of the variable *d* can become a bottleneck, since the underlying Markov chain analysis is a computationally intensive process. An option would be to calculate the *d* values in advance and store them as a mapping structure on the client, with software updates including a recalculation of the *d* should such a need arise.

More precisely, the client needs to perform the computation of the matrices *PM*, Y, and Π followed by the sum $E[\Pi_{l}]$ as explained in Section 3 only once.

Furthermore, by allowing a user to specify an approximation of the size of the malicious cluster (based on how pessimistic the user is about the state of the network), the user can lower the execution time by specifying a smaller number of assumed malicious nodes (in the Aurora paper, the assumed number of malicious nodes is always l=⌈|S|/2⌉−1).

This would primarily reduce *l*, which in turn would reduce the complexity of calculating *d* as expressed by Equation (6), as well as the sum expressed by Equation (4), resulting in a smaller *d*.

Potential improvement would be to cache the traversed set of nodes, which means that the client can query the endpoints of the traversed nodes without having to re-run the algorithm.

Consequently, the client does not have to perform a *walk* every time a transaction inclusion verification is required, as a *walk* requires the exchange of 2∗d messages, as expressed by Equation (7). Instead, the honest node that was the output of the previous walk can be contacted again.

For a more detailed discussion on the topic, see Section 6.

In summary, the algorithm is more efficient for solutions that allow faster discovery of new nodes, such as Bitcoin, where $\bar{\left|\mathrm{APRES}\right|}=1000$ and can be run on resource-constrained devices such as smartphones, where storing the entire chain or header chain is not an option.

### Comparison to Other Clients

In order for the Aurora client to run in resource-constrained environments, our solution aims to run with a low amount of disk space and RAM. Table 4 compares different clients with the Trinity client enhanced with the Aurora module. The data on our client were measured when the client was running in light mode with Feature 2 enabled (i.e., transaction verification mode). This means that all unnecessary components were disabled during initialization. No further code optimizations were made in favor of reducing the memory consumption.

The results for disk space consumption yield a competitive value of 1 MB, which is mainly used for network discovery-related data, and approximately 800 MB of RAM is used at runtime, out of which only 0.31 MB is used by the Aurora component. Compared to Trinity (full), Trinity (light), geth (fastsync) and geth (light), the main advantage of our client is that it uses significantly less storage. Compared to BlockQuick, our client consumes more storage and RAM, but BlockQuick requires the presence of additional data structures which require the introduction of disruptive backward-incompatible changes in the network (a hard fork), while our client does not. Compared to Infura, the client requires more storage and significantly more RAM. However, Infura is a trusted solution, while our client is an trustless solution. Our Proof of Concept implementation is capable of running on constrained devices [36] with as little RAM as the Raspberry Pi 2 Model B (1 GB RAM) (https://www.raspberrypi.org/products/raspberry-pi-2-model-b/, accessed on 11 February 2022).

## 6. Discussion and Future Work

This work has clearly indicated possible starting points for future work. Since we have no insight into the absolute state of the Ethereum network, and introducing malicious actors into a production network is non-trivial from many perspectives, we will shift our evaluation to simulation tools as part of our verification procedure.

Since execution time has a linear dependence on the assumed number of malicious nodes in the network, we will work towards creating a complexity analysis that is a function of the assumed number of malicious nodes.

Although the algorithm indirectly depends on the use of the hypergeometric distribution, the output of the algorithm is not probabilistic, but rather a derivative of an underlying probability. One could argue that the modification of the algorithm should provide a unique probabilistic output. Furthermore, the computation of the distance parameter *d* is computationally intensive, which can be solved by precomputing the values and storing them on the client running the algorithm.

Moreover, our experiments conducted on the mainnet show that an available peer slot is a scarce resource. This problem can be addressed in three different ways. First, a node executing the algorithm can query peers in parallel and filter out unreachable peers, which reduces the execution time. Second, an additional communication port for querying the peer neighbor list can be opened at each node network-wide. This extension would generate a low network traffic, but would create DoS and DDoS vulnerabilities as a byproduct. A practical solution to such vulnerabilities is for a remote full node to increase exponentially the period between two responses to a single node. Third, as the number of public and reachable nodes is expected to increase over time, the problem will mitigate itself to some degree.

Finally, full nodes with the ability to serve light clients are also a scarce resource on the mainnet. While this problem affects our client, it is part of a broader problem that affects all light clients. We argue that current chain head requests and peer list requests and responses are already supported by a large majority of nodes in the network and can be used by our algorithm to achieve a leaner initial ledger download. If all nodes do not provide responses required to execute the transaction inclusion verification method, our solution could be extended to include an incentive mechanism. However, the design of an incentive mechanism for this purpose is beyond the scope of this work.

## 7. Conclusions

Client execution on DLT networks such as Bitcoin and Ethereum is becoming increasingly resource intensive. As a result, most light or remote clients sacrifice decentralization for convenience. Moreover, the initial blockchain download can be considered as a point of centralization, since it depends on the assumption that a set of well-known nodes is both available and does not exhibit Byzantine or malicious behavior. This paper explores the scenario in which this assumption does not hold, and proposes a method to mitigate the impact of malicious nodes in DLT networks. In particular, our solution is stochastic in nature and allows a client to securely download the header chain and efficiently verify the inclusion of transactions in a block in the presence of malicious actors.

As part of our contribution in this paper, we evaluate the Aurora algorithm proposed in our previous work by implementing the features of the algorithm in the Ethereum Trinity client. We discuss the updated Trinity client architecture, followed by an analysis of client performance when executing the Aurora algorithm on the Ethereum mainnet. We compare the resource consumption of the Aurora-Trinity other state-of-the-art light clients.

Our results clearly demonstrate that the integration of the Aurora algorithm into existing DLT solutions is feasible and that the main advantage of our client is its compatibility with the current Ethereum network, which means that no major changes within the network are required to support the algorithm.

The results of the conducted experiments show that our Proof of Concept solution is capable of running on devices such as a Raspberry Pi 2 Model B and that the Aurora component itself in the Trinity client consumes a competitive 0.31 MB of RAM and 1 MB of additional storage, which makes it suitable for resource-constrained environments.

However, there are still practical limitations of the algorithm due to the non-ideal state of the Ethereum network. Since the execution time depends on the average peer list response size, small average peer list responses increase the execution time. This problem can be addressed with parallelization of client requests. Moreover, not many full nodes currently have the capabilities or incentive to serve light clients. While this fact is an obstacle to run our client, it is part of a larger problem that is beyond the scope of this paper.

Driven by the findings in this paper, we will focus our future work towards a deeper evaluation of our algorithm by the use of Monte Carlo simulations within a strictly controlled environment, as the introduction of malicious nodes on the Ethereum network is both impractical and unethical. We will also refine our model to provide a clear probabilistic interpretation coupled with a complexity analysis in an effort to improve efficiency and execution time, followed by the identification of specific DLT solutions that will benefit the most from our solution.

In summary, integrating our solution with existing DLT clients is feasible and improves the security and decentralization properties of DLT solutions, synergizes with some of the existing related work, reduces resource consumption in the presence of malicious and Byzantine actors, and is applicable to both consumer-grade hardware and resource-constrained devices.

## Figures and Tables

**Table 1:** Mapping of Aurora requirements to Ethereum P2P protocol messages.

Description	Aurora	NDP v4	LES v2	ETH v63
Neighbors of a peer	APREQ	FindNode (0 × 03)	—	—
Head hash	head	—	Status (0 × 00)	Status (0 × 00)
Transaction proof	proof	—	GetProofsV2	—

**Table 2:** Results of RLPx connection attempts with peers in the mainnet Ethereum network.

Result	ETH	LES
Successful	12 (0.83%)	1 (0.02%)
HandshakeFailureTooManyPeers	543 (37.65%)	1061 (22.07%)
NoMatchingPeerCapabilities	293 (20.31%)	3088 (64.24%)
TimeoutError	134 (9.29%)	402 (8.36%)
UnreachablePeer	52 (3.60%)	169 (3.51%)
PeerConnectionLost	351 (24.34%)	12 (0.25%)
WrongNetworkFailure	30 (2.08%)	24 (0.50%)
HandshakeFailure	—	49 (1.01%)
WrongGenesisFailure	27 (1.87%)	—
MalformedMessage	—	1 (0.02%)

**Table 3:** Execution times for the calculation of *d* based on various input parameters, with network size = 7000. *M*—assumed malicious cluster size. Execution time was extrapolated from the average mainnet hop duration of 7 s and optimal *d* reduction at runtime.

M	$\bar{\left|\mathrm{APRES}\right|}$	*d*	*d* Calculation Time	*walk* Duration
3499	16	3819.07	1.16 h	3.71 h
500	16	2969.14	1.36 min	2.88 h
500	48	987.78	1.64 min	57.61 min
100	16	2267.51	3.64 s	2.20 h
100	18	754.43	4.11 s	43.83 min

**Table 4:** Approx. specifications for various Ethereum clients compared to the Trinity Aurora client.

Client	Trust	Storage	RAM	Breaking Changes
Trinity Aurora	Trustless	∼1 MB	∼800 MB	no
Trinity (light)	Trustless	>1.2 GB	∼800 MB	no
Trinity (full)	Trustless	>200 GB	∼2 GB	no
geth (fastsync)	Trustless	>200 GB	∼1 GB	no
geth (light)	Trustless	>1.2 GB	∼150 MB	no
BlockQuick	Trustless	∼20 KB	∼50 KB	yes
Infura	Gateway (Trusted)	∼4 KB	∼10 KB	no
